# Effect of two teaching methods on nursing students' acquisition of patient-centered communication competence in older people care: a cluster randomized trial

**DOI:** 10.3389/fpubh.2024.1510620

**Published:** 2024-12-23

**Authors:** Alda Elena Cortés-Rodríguez, María Mar López-Rodríguez, Pablo Roman, José Granero-Molina, Cayetano Fernández-Sola, José Manuel Hernández-Padilla

**Affiliations:** ^1^Department of Nursing, Physiotherapy and Medicine, University of Almería, Almería, Spain; ^2^Health Sciences Research Centre, University of Almería, Almería, Spain; ^3^Faculty of Health Sciences, Universidad Autónoma de Chile, Santiago, Chile

**Keywords:** generation Z, nursing, older people, patient-centered communication, role-play, simulation, standardized patient

## Abstract

**Introduction:**

Patient-centered communication is an essential skill in nursing, particularly in the care of older adult patients. However, generation Z nursing students, who primarily communicate through digital platforms, face unique challenges in adapting to traditional face-to-face communication with older adults. As a result, there is a need for teaching methods that align with this generation's learning style to enhance their communication skills. This study aimed to compare the effectiveness of two teaching methods—standardized patient simulation and role-play—on nursing students' acquisition of patient-centered communication competence in older people care.

**Methods:**

A controlled cluster-randomized trial was conducted with 124 nursing students, divided into eight teaching groups. Students participated in either a standardized patient simulation or a role-play workshop, each consisting of a 1.5-h online module and a 1.5-h face-to-face session. The three components of patient-centered communication competence—knowledge, skills, and self-efficacy—were assessed using simulated scenarios at pre-test, post-test, and 6-week follow-up. Between-group and within-group differences were measured based on the number of students who achieved competence.

**Results:**

Both interventions significantly improved students' knowledge, skills, and self-efficacy in patient-centered communication between pre- and post-tests, with improvements maintained at follow-up. No significant differences were found between the two methods.

**Conclusions:**

Both standardized patient simulation and role-play are effective in enhancing patient-centered communication competence in older people care. However, neither method was found to be superior in teaching knowledge, skills, or self-efficacy.

## 1 Introduction

Older people, defined as aged 65 and over, account for more than 9% of world population (1). The increase in life expectancy, as well as medical advances, has stimulated the growth of this demographic group and, therefore, the demand for economic, social, and health resources (2). Older people often have several chronic diseases, and they experience a decrease in their abilities that exacerbates their frailty and vulnerability (3). This situation leads to an increased use of health system, involving frequent contact between nurses and these patients.

Nursing care for older people involves a set of specialized competences to meet the needs of this group and to provide holistic and quality care. Patient-centered care seems to be the best approach to care for older people since it promotes their participation in healthcare and it fosters their autonomy, improving their quality of life (3). This patient-centered care is based on a good nurse-patient communication that allows understanding of the needs of these patients and stimulating decision-making according to their principles (4). However, several studies have shown that nurses often report difficulties communicating with older people and managing their emotions appropriately (2). Furthermore, attitudes toward older people have traditionally been negative, related to strong social stereotypes about aging (5). Beyond this, generational differences between older people and new generations of nurses seem to increase nurse-patient communication difficulties (6).

Currently, five generational cohorts coexist in our society, and they understand the world from quite different perspectives. The newest generation of nursing students belongs to what has been called generation Z. People in this generation have always had the Internet and smartphones present in their lives. In this way, social media is their normal way to communicate, using visual content and messaging apps to be constantly connected (6). This way of communication is completely different from previous generations (7), so, when nursing students need to talk and communicate with older people, they report having difficulties. They report problems starting a conversation or asking basic questions. Furthermore, they do not feel qualified to adjust the information to these patients, determine their emotional concerns and needs, and to support those who are experiencing a problematic health situation (8, 9).

All qualified nurses should achieve general, transversal, and specific competencies. General competencies refer to broad skills required in various contexts, such as critical thinking and problem-solving. Transversal competencies are essential skills that cut across different areas of professional practice, including communication, teamwork, and leadership. Specific competencies are specialized skills tailored to a particular area of practice, such as geriatric care or pediatric nursing. Among these, patient-centered communication is defined as a transversal competence focused on transferring information through verbal and non-verbal behaviors to establish a therapeutic relationship with patients and their families (10, 11). Being competent implies presenting the ability to use knowledge, skills, and attitudes appropriately (12, 13). Moreover, it is known that, along with knowledge and skills, students should have a high level of self-efficacy, which is defined as the belief in the ability to act effectively (14). Globally, patient-centered communication is conceived as a core component of nursing training, therefore several teaching methods have been developed to help nursing students to acquire competence in terms of knowledge, skills, and self-efficacy. Nursing students, given their generational characteristics, learn better through observation and practice, rather than with lectures (6, 15). Thus, methodologies such as standardized patient simulation or roleplay have been shown to foster the acquisition of this competence because these strategies are based on experiential learning activities (16, 17).

Standardized patient simulation is considered a good method to increase patient safety and to promote patient-centered communication. This strategy offers a high level of realism because it involves actors performing as patients in different clinical scenarios (18). However, this method is applied in small teaching groups, and since its implementation involves more complex scenarios and trained actors, it usually implies a high investment of resources (18–21). On the other hand, roleplay requires less time and financial investment since it can be applied in larger groups in which students play different roles to solve a specific clinical situation (19, 20, 22). However, this method assumes less realism and students usually report not feeling comfortable doing the performances (23).

Currently, few studies have compared the effectiveness of these methods in acquiring patient-centered communication skills (21, 24–26). Studies using standardized patient simulation found an improvement in skills and self-efficacy (26, 27). On the other hand, some research showed that roleplay leads to an improvement in communication skills compared to lectures and discussion groups (17, 25, 28). However, to the best of our knowledge, the effects of these methods in the acquisition of patient-centered communicative competence in older people care have not been compared. The aim of this study was to compare the effects of two teaching methods (standardized patient simulation vs. roleplay) on the acquisition of patient-centered communication competence in the care of older people among nursing students that belong to generation Z.

## 2 Materials and methods

### 2.1 Design

A controlled clustered randomized trial design was used.

### 2.2 Sample and recruitment

**Figure 1:**
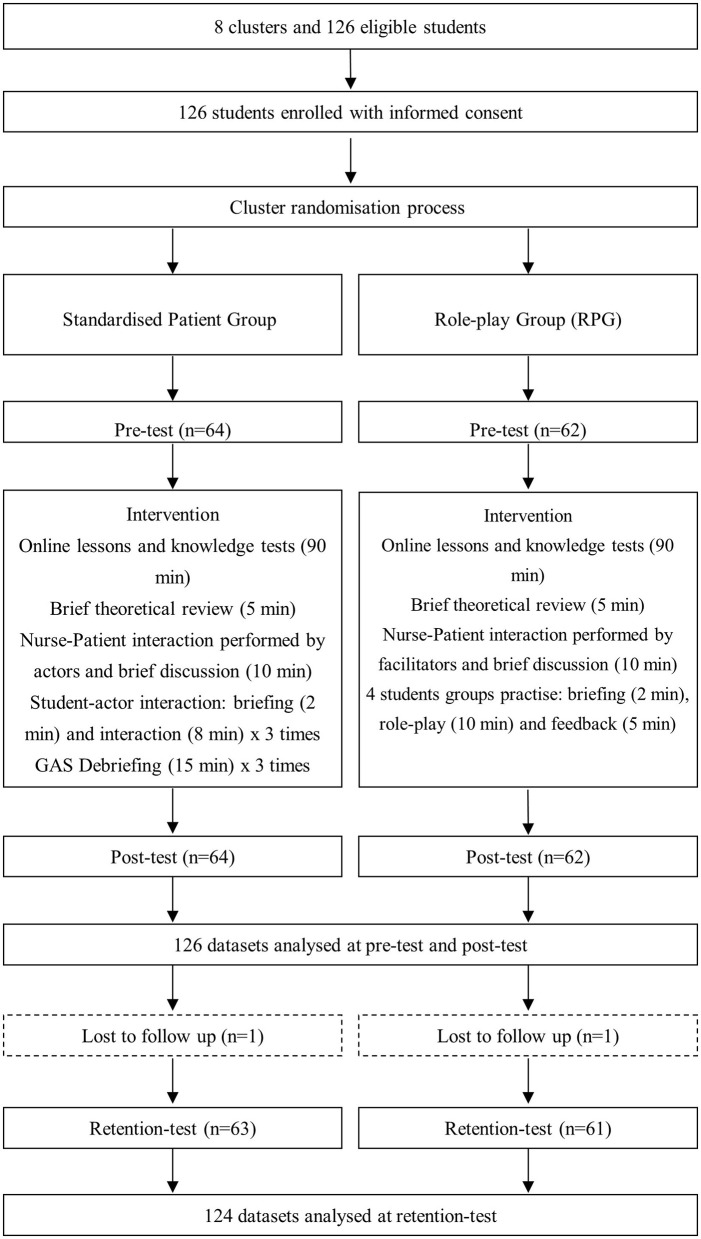
Flowchart for study design.

Students' clusters were divided into two training groups: the standardized patient simulation group or the role-play group (Figure 1). The research was carried out at [Universidad de Almería] between September 2017 and February 2018 and is related to another previously published study that had the same sample of participants (29).

Participants' inclusion criteria were: 1. to be at least 18 years old, 2. to belong to Generation Z, 3. to be enrolled in the “Older adults Care” module of the nursing degree at the [Universidad de Almería], and 4. not to have received any formal training in patient-centered communication. G-power^®^ version 3.1.9.4 was used to calculate the sample size and power analysis was performed based on the differences between two proportions. In order to achieve a 95% confidence level and 80% power to detect statistically significant differences (*p* < 0.05), the estimated sample size was 56 subjects per group. Since all students enrolled in the “Older adults Care” module volunteered to participate and considering potential losses to follow-up, a total sample of 126 was initially recruited for the study. The sociodemographic characteristics gathered were age, sex, and educational level.

### 2.3 Data collection

Randomization was carried out at a cluster level. Eight teaching groups of 16–18 students were previously established by the faculty administrative staff. Organizationally, when a teaching group is attending a face-to-face session for a given module, the rest of the groups are also attending sessions for other modules; hence why randomization could not be performed at an individual level. For this study, the teaching groups were considered as clusters and each cluster was blindly assigned a numerical code [1–8]. Using Research Randomizer^®^ v.4.0, each cluster was randomly allocated to either the standardized patient simulation group or the role-play group.

The summary of the teaching protocol can be seen in Figure 1. Both groups completed a 3-h workshop on patient-centered communication in older people care. This workshop was comprised of a 1.5 h online module which included video-recorded lessons and knowledge tests, and a 1.5 h face-to-face session based on standardized patient simulation or role-play. The face-to-face session started with a brief review of the main concepts about patient-centered communication in older people care based on the SAGE & THYME communication model. This model emphasizes building rapport, understanding patients' needs, and responding empathetically, which is key for fostering effective communication (30). Students then observed and analyzed a nurse-patient interaction performed by two actors (standardized patient simulation) or two facilitators (role-play). After that, in the standardized patient simulation group, the training was carried out with an actor who played an older patient in three different scenarios. Thus, a student interacted with the actor on each setting and their peers observed them, to end up participating in a debriefing as recommended by the International Nursing Association for Clinical Simulation and Learning (31). The Gather-Analyze-Summarize (GAS) method was used on the debriefing to facilitate collecting information, reflect on it, and summarize the lessons learnt (32). In the role-play group, groups of four students were formed to work on four case studies. In each group, one student played the nurse role, another student acted as an older patient and the remaining students observed the interaction and gave feedback about those things that went well, those that could have been improved and a positive comment to take forward. In each case study, students exchanged roles so that they could practice the nurse role.

To ensure consistency and minimize bias, the same facilitator delivered all the training workshops. This facilitator had completed postgraduate courses on clinical simulation and had previous experience using role-play, ensuring the necessary expertise to guide both types of interventions. Additionally, all workshops followed a standardized structure, including identical durations for theoretical reviews, observation activities, and interactive components. In the standardized patient simulation group, the facilitator moderated the debriefing sessions, while in the role-play group, provided consistent feedback to participants. These measures were implemented to maintain intervention fidelity throughout the study.

### 2.4 Instruments

The competence in patient-centered communication in older people care was individually assessed before (pre-test), immediately after completion of the workshops (post-test) and 6 weeks after the intervention (retention test). To test psychomotor skills, students had to interact with a previously trained actor in a simulated scenario while a researcher observed their performances. All assessments were videotaped and two researchers separately marked participants' interactions.

The level of knowledge on patient-centered communication in older people care was assessed with the Person-Centered Communication subscale of a multiple-choice questionnaire (PCC-MCQ) (33). The PCC-MCQ comprised eight questions about the SAGE&THYME model (30) with five options and only one correct answer, including an “I don't know” answer. Its construct validity was good (ICC = 0.52).

The level of self-efficacy was assessed with the “Person-centered Communication Self-Efficacy Scale” (PCC-SES) of the “Clinical Communication Self-Efficacy Toolkit” (34). PCC-SES comprised 17 items rated on a scale 0–100, from “I'm sure I can't do it” to “I'm sure I can do it.” Its internal consistency was very good (Cronbach α = 0.93).

Psychomotor skills were assessed using the Person-Centered Checklist (PCC-Checklist) (33). The PCC-Checklist comprised 17 items on the skills needed to efficiently communicate with older people. Using a rubric, the items were rated on a scale of 0–5, from “not competent” to “fully competent.” Its internal consistency was excellent (Cronbach α = 0.95).

### 2.5 Outcome measures

In reference to the level of knowledge, following marking standards in the environment where the study was performed and taking into account similar studies' benchmarks, it was determined that it was necessary to achieve a score equal to or >70% on the PCC-MCQ (29, 35).

On self-efficacy, a score equal to or >70% was considered sufficient, as recommended by other authors in similar studies (29, 36, 37).

Regarding psychomotor skills, an average score of 3 points or more was considered adequate (36).

Finally, participants were considered to have achieved competence in patient-centered communication in older people care when they scored ≥70% on PCC-MCQ, ≥3 points on PCC-Checklist, and ≥70% on PCC-SES.

### 2.6 Data analysis

Statistical analysis of the data was performed using IBM^®^ SPSS^®^ v.25 for Windows. Firstly, a descriptive analysis of sociodemographic variables was conducted. The key baseline demographic variables were compared between the groups using independent *t*-tests for continuous data and chi-squared tests for categorial data. To know the effect of the interventions, the frequency and percentage of students who reached the benchmark in each component, as well as for competence in patient-centered communication in older people, were calculated at the pre-test, post-test, and 6-weeks follow-up. Between-subjects differences were assessed using the chi-Squared test. Meanwhile, within-subject differences were evaluated using the McNemar test. For these analysis, *p*-value < 0.05 was considered statistically significant.

Finally, a generalized estimating equation (GEE) analysis with logit link function was used to compare the differences in the counts and proportions of students who achieved competency within each intervention. In this case, Bonferroni correction was applied, and differences were considered statistically significant if *p*-values < 0.025.

### 2.7 Ethical considerations

The ethics committee of [Universidad de Almería] granted permission to conduct the study before beginning the recruitment phase. Eligible participants were provided with written detailed information about the study's aims and procedures, and those volunteering to participate signed an informed consent before enrolling in the study. Moreover, they were informed about their right to withdraw from the investigation without any academic consequences. All data were processed in accordance with the European Data Protection Legislation (38). The study was not registered.

## 3 Results

### 3.1 Sample characteristics

Table 1 shows the main demographic characteristics of the participants.

**Table 1:** Demographic characteristics of participants.

	**SPG (*N* = 64)**	**RPG (*N* = 62)**	**Total sample (*N* = 126)**	***t*-test**	***p*-value**
	***M*** ±**SD**	***M*** ±**SD**	***M*** ±**SD**		
**Age (years)**	22.77 ± 6.70	22.29 ± 6.03	22.53 ± 6.36	−0.44	0.66
	***n (%)***	***n (%)***	***n (%)***	*X* ^2^	***p-*** **value**
**Gender**				0.54	0.46
Female	51 (79.7)	46 (74.2)	97 (77.0)		
Male	13 (20.3)	16 (25.8)	29 (23.0)		
**Education level**				2.48	0.48
Upper secondary	62 (96.9)	61 (98.4)	123 (97.6)		
Degree	2 (3.1)	1 (1.6)	3 (2.4)		

SPG, Standardized Patient Simulation Group; RPG, Role-Play Group; M, mean; SD, Standard Deviation; X^2^, Chi-Squared test.

The sample consisted of 77% (*n* = 97) female participants belonging to generation Z with a mean age of 22.53 ± 6.36 years old. Regarding the level of education, 97.6% (*n* = 123) had completed upper secondary education before entering the nursing degree (Table 1).

### 3.2 Intervention outcomes

The number of participants who achieved the safety benchmarks for knowledge, self-efficacy, and psychomotor skills of patient-centered communication in older people care competence, as well as general competence, in both intervention groups in the pre-test, post-test, and retention measures, is collected on Table 2. GEE analysis did not show statistically significant differences over time between groups for any of the variables studied (*p* > 0.05).

**Table 2:** Counts (proportions) of participants who achieved the benchmark for all variables measuring competence in patient-centered communication in older people care and GEE analysis.

	**SPG**			**RPG**			**Time vs. int**.	
	**Pre-test** ***n*** = **64**	**Post-test** ***n*** = **64**	**Retention-test** ***n*** = **63**	**Pre-test** ***n*** = **62**	**Post-test** ***n*** = **62**	**Retention-test** ***n*** = **61**	**B (95% CI)**	***p*** **-value** ^a^
**Knowledge**								
≥70% of PCC-MCQ answered correctly	7 (11%)	45 (70%)	38 (59%)	10 (16%)	38 (61%)	37 (61%)	−0.42 (0.30-1.46)	0.31
**Self-efficacy**								
≥70% achieved in PCC-SES	35 (55%)	54 (84%)	46 (73%)	34 (55%)	53 (85%)	52 (85%)	0.67 (0.69-5.60)	0.21
**Communication skills**								
≥3 points achieved in PCC-Checklist	21 (33%)	60 (94%)	59 (64%)	17 (27%)	54 (87%)	54 (88%)	−0.15 (0.14-5.36)	0.87
**Patient-centered communication competence**								
Overall competence achieved^b^	1 (2%)	35 (55%)	27 (43%)	2 (3%)	33 (53%)	32 (52%)	−0.50 (0.27-1.38)	0.23

SPG, Standardized Patient Simulation Group; RPG, Role-Play Group; PCC-MCQ, Person-Centered Communication Multiple-Choice Questionnaire; PCC-Checklist, Person-Centered Checklist; PCC-SES, Person-Centered Communication Self-Efficacy Scale; CI, Confidence Interval.

^a^GGE analysis: p-value in time vs. intervention group interaction. Significance is reached at 0.025, according to the Bonferroni correction = 0.05/2.

^b^Patient-centered communication competence: ≥70% of PCC-MCQ answered correctly, ≥70% achieved in PCC-SES; and ≥3 points achieved in PCC-Checklist.

Learning improvement from pre-test to post-test was compared for both interventions using the McNemar test (Table 3). The results showed a statistically significant improvement in the three components of the competence as well as on overall patient-centered communication in older people care competence in both intervention groups (*p* < 0.05).

**Table 3:** Counts (proportions) of dichotomous patient-centered communication competence components per group for pre-test and post-test.

	**SPG**			**RPG**			**SPG vs. RPG pre-test**	**SPG vs. RPG post-test**
	**Pre-test** ***n*** = **64**	**Post-test** ***n*** = **64**	***p*** **-value** ^a^	**Pre-test** ***n*** = **62**	**Post-test** ***n*** = **62**	***p*** **-value** ^a^	***p*** **-value** ^b^	***p*** **-value** ^b^
**Knowledge**								
≥70% of PCC-MCQ answered correctly	7 (11%)	45 (70%)	< 0.001	10 (16%)	38 (61%)	< 0.001	0.39	0.29
**Self-efficacy**								
≥70% achieved in PCC-SES	35 (55%)	54 (84%)	< 0.001	34 (55%)	53 (85%)	< 0.001	0.99	0.86
**Communication skills**								
≥3 points achieved in PCC-Checklist	21 (33%)	60 (94%)	< 0.001	17 (27%)	54 (87%)	< 0.001	0.51	0.20
**Patient-centered communication competence**								
Overall competence achieved^c^	1 (2%)	35 (55%)	< 0.001	2 (3%)	33 (53%)	< 0.001	0.54	0.87

SPG, Standardized Patient Simulation Group; RPG, Role-Play Group; PCC-MCQ, Person-Centered Communication Multiple-Choice Questionnaire; PCC-Checklist, Person-Centered Checklist; PCC-SES, Person-Centered Communication Self-Efficacy Scale.

^a^McNemar test.

^b^Chi-squared test.

^c^Patient-centered communication competence: ≥70% of PCC-MCQ answered correctly, ≥70% achieved in PCC-SES; and ≥3 points achieved in PCC-Checklist.

Table 4 shows the results of the comparison of the level of knowledge, self-efficacy, skills, and overall patient-centered communication in older people care competence between the post-test and retention test. The McNemar's test results showed a decrease in knowledge and skills, as well as on overall competence in the standardized patient simulation group, although these differences were not statistically significant (*p* > 0.05). Regarding role-play group, a decrease in the success rates was also observed on self-efficacy and skills components and on overall competence, although the differences were not statistically significant either (*p* > 0.05).

**Table 4:** Counts (proportions) of dichotomous patient-centered communication competence components per group for post-test and retention-test.

	**SPG**			**RPG**			**SPG vs. RPG retention-test**
	**Post-test** ***n*** = **63**	**Retention-test** ***n*** = **63**	***p*** **-value** ^a^	**Post-test** ***n*** = **62**	**Retention-test** ***n*** = **61**	***p*** **-value** ^a^	***p*** **-value** ^b^
**Knowledge**							
≥70% of PCC-MCQ answered correctly	45 (70%)	38 (59%)	0.14	38 (61%)	37 (60%)	1	0.97
**Self-efficacy**							
≥70% achieved in PCC-SES	53 (84%)	46 (73%)	0.09	52 (85%)	52 (85%)	1	0.09
**Communication skills**							
≥3 points achieved in PCC-Checklist	59 (94%)	59 (94%)	1	53 (87%)	54 (89%)	1	0.32
**Patient-centered communication competence**							
Overall competence achieved^c^	35 (55%)	27 (42%)	0.12	33 (53%)	32 (52%)	1	0.29

SPG, Standardized Patient Simulation Group; RPG, Role-Play Group; PCC-MCQ, Person-Centered Communication Multiple-Choice Questionnaire; PCC-Checklist, Person-Centered Checklist; PCC-SES, Person-Centered Communication Self-Efficacy Scale.

^a^McNemar test.

^b^Chi-squared test.

^c^Patient-centered communication competence: ≥70% of PCC-MCQ answered correctly, ≥70% achieved in PCC-SES; and ≥3 points achieved in PCC-Checklist.

Lastly, the differences in the proportion of participants who achieved the benchmark for all the components and overall competence at pre-test and retention-test for both groups are shown in Table 5. Statistically significant differences between pre-test and retention-test measures were found in the three components of the competence as well as in overall patient-centered communication in older people care competence, regardless of the intervention (*p* < 0.05).

**Table 5:** Counts (proportions) of dichotomous patient-centered competence components per group for pre-test and retention-test.

	**SPG**			**RPG**		
	**Pre-test** ***n*** = **64**	**Retention-test** ***n*** = **63**	**p-value** ^a^	**Pre-test** ***n*** = **62**	**Retention-test** ***n*** = **61**	**p-value** ^a^
**Knowledge**						
≥85% of PCC-MCQ answered correctly	7 (11%)	38 (59%)	< 0.001	10 (16%)	37 (60%)	< 0.001
**Self-efficacy**						
≥70% achieved in PCC-SES	34 (54%)	46 (73%)	0.004	34 (56%)	52 (85%)	< 0.001
**Communication skills**						
≥3 points achieved in PCC-Checklist	21 (33%)	59 (94%)	< 0.001	17 (28%)	54 (89%)	< 0.001
**Patient-centered communication competence**						
Overall competence achieved^b^	1 (2%)	27 (42%)	< 0.001	2 (3%)	32 (52%)	< 0.001

SPG, Standardized Patient Simulation Group; RPG, Role-Play Group; PCC-MCQ, Person-Centered Communication Multiple-Choice Questionnaire; PCC-Checklist, Person-Centered Checklist; PCC-SES, Person-Centered Communication Self-Efficacy Scale.

^a^McNemar test.

^b^Patient-centered communication competence: ≥70% of PCC-MCQ answered correctly, ≥70% achieved in PCC-SES; and ≥3 points achieved in PCC-Checklist.

When comparing the success rates of the standardized patient simulation group and the role-play group applying the Chi-square test, no statistically significant differences were observed in the pre-test, post-test, or retention-test on any of the three components or in the overall patient-centered communication in older people care competence.

## 4 Discussion

The purpose of this study was to describe and compare the effects of two teaching methods (standardized patient simulation and roleplay) on the acquisition of patient-centered communication competence in older people care amongst nursing students that belong to generation Z. The number of students achieving a good level of competence was higher after applying both methods, leading to increased levels of knowledge, skills, and self-efficacy. However, no statistically significant differences were found between both methods for any of the studied variables.

Roleplay and standardized patient simulation have been shown to be effective methods for teaching communication skills to nursing students (17, 21, 25, 39). The combination of lectures, discussion groups, and the assumption of different roles to solve case studies have proved to improve students' self-efficacy, as well as their skills and knowledge of patient-centered communication (17, 25, 28). Along with these results, the use of standardized patients in more realistic scenarios and the subsequent debriefing with peers have also demonstrated good learning results in this competence (24, 26, 27). However, this study appears to be the first among nursing students of generation Z to compare the effectiveness of these two teaching methods in the acquisition of patient-centered communication competence in older people care.

Regarding knowledge, the results showed no statistically significant differences between standardized patient simulation and roleplay. These data are consistent with a study by Quail et al. (40) among speech pathology students, which showed no differences in communication knowledge between standardized patient simulation and virtual patient simulation. These results could be explained by the idea that these methods promote the development of helpful reflections and a greater awareness about what is important to communicate effectively with patients (41). In addition, discussion groups, observation, and feedback from peers or through debriefing may have promoted self-reflection and self-assessment and may have contributed to strengthen participants' knowledge (42–45).

In terms of self-efficacy, although there was a higher number of participants who reached a good level of competency between measures, no statistically significant differences were found when comparing both methods. These results are consistent with previous studies that applied standardized patient simulation (46, 47) and roleplay (17, 25, 48). Overall, participants obtained high levels of self-efficacy at pre-test, post-test, and retention measures which demonstrate they had good confidence in their communication skills with older people. Both interventions provided a safe environment where mistakes did not lead to serious consequences, so this could have made participants more confident when interacting with older adults (49, 50). Additionally, working in groups and receiving feedback on their performance could also facilitate this environment of security and trust (17). On the other hand, the fact that no differences were found between both interventions could be supported by the short duration of the workshops, which limited repetition, a key learning facilitator (17, 48, 51). However, it seems that both interventions allowed participants to become aware of their abilities and the relevance of the competence they were working on, which could have influenced their scores (51–53). Furthermore, the participants had no previous experience communicating with older people and they were going to start a clinical placement, so this could have resulted in greater motivation and an increase on their self-efficacy (17, 54).

Regarding skills, the results also showed a statistically significant increase in the number of participants who reached a good level of skills after both standardized patient simulation and roleplay, although no statistically significant differences were found between both methods. These results agree with previous studies that have applied some of these interventions (47, 48, 55, 56). This improvement in patient-centered communication skills could be explained by the fact that both interventions had a strict structure, with a very similar approach (44, 51). Additionally, the participants belonged to generation Z. In this way, the use of these experiential methods based on the assumption of different roles and modeling could have benefited the acquisition of these skills (44, 57). Lastly, these learning methods included student-lecturer interaction and feedback from classmates, both learning facilitators which could have promoted a better understanding of their roles and the way they were performing (17, 44).

Finally, our results showed that the success rates in knowledge, skills, and self-efficacy were higher after applying standardized patient simulation and roleplay. This implies that, regardless of the method, these generation Z participants were able to communicate better with older people, reducing the difficulties reported in previous research. Thereby, these outcomes could be explained by several factors. On the one hand, modeling, skill performance, and feedback were basic components of both interventions. These activities reduce the cognitive demands that learning such skills imposes on students (36) and promote the integration of competence (37, 58). Furthermore, this approach is based on self-directed training, as well as self-assessment, which could have increased motivation and could have made students more aware of their mistakes, promoting changes in their behaviors (37, 59). On the other hand, both interventions were carried out in scenarios that did not have the same characteristics as a clinical setting or a gerontology unit, and this could have made the participants feel uncomfortable, reducing the learning results (21, 25, 40, 46). In addition, a limited number of scenarios were used due to the short duration of the workshops, which could directly have influenced participants' learning outcomes by reducing the chances of practicing and observing their peers (17, 40, 48, 51).

### 4.1 Limitations

To the best of our knowledge, this is the first study that has sought to compare the implementation of two teaching methods in acquiring patient-centered communication competence in older people care. However, there are some limitations that may influence the interpretation of the results. First, the sample in this study came from a local university, with specific characteristics and met very specific inclusion criteria. This means that the outcomes cannot be generalized to other populations. Second, even though the interventions were well-defined, since they included a combination of activities, it is not possible to determine the effect of each activity on the outcomes. Third, the participants had no previous experience in standardized patient simulation, so they could have felt uncomfortable and nervous with this method, affecting their scores (17, 46, 48). Fourth, no formal pre-briefing was planned before starting the interventions, and evidence suggests this is necessary to establish a safe environment. Therefore, this could have affected participants performance (60). Fifth, we set the benchmark at 70% for all variables according to the marking systems in our environment, because there was no defined evidence-based benchmark to consider a sufficient level of competence in knowledge, self-efficacy, and skills. Finally, it is not possible to know how the levels of this competence have been maintained since only a 6-week follow-up was performed.

### 4.2 Implications

This study carries significant implications across nursing research, education, and practice. In research, it provides empirical support for the effectiveness of role-play and simulation in enhancing patient-centered communication in gerontology. This encourages further exploration of innovative pedagogical approaches. In education, it advocates for the incorporation of these methods into nursing curricula, equipping students with vital communication skills for older people care. In nursing practice, it underscores the importance of patient-centered, generational-aware communication, potentially elevating the quality of care for older adults in an aging population.

## 5 Conclusion

Standardized patient simulation and roleplay have proved to be two good methods for teaching patient-centered communication competence in older people care among nursing students that belong to generation Z. These interventions lead to a higher number of students acquiring and retaining knowledge, skills, and self-efficacy in this competence, although the results showed no superiority of any of these methods. This implies that the use of these methods allows students to overcome communication problems with older people and provide comprehensive care that improves the quality of life of these people. Future studies should focus on measuring participants' stress and anxiety levels of participants because these factors can influence the performance of students. Moreover, future research should conduct larger studies, including long-time interventions and medium-term follow-ups, to know the real impact of these interventions on patient-centered communication competence.

## Data Availability

The raw data supporting the conclusions of this article will be made available by the authors, without undue reservation.

## References

[1] Banco Mundial. Población de 65 años de edad y más (% del total) (2022). Available at: https://datos.bancomundial.org/indicator/SPPOP65UPTOZS?end=2019&start=1960 (accessed March 18, 2023).

[2] Adibelli D Kiliç D. Difficulties experienced by nurses in older patient care and their attitudes toward the older patients. Nurse Educ Tod. (2013) 33:1074–8. 10.1016/j.nedt.2012.04.002 22542986

[3] Hafskjold L Sundler AJ Holmström IK Sundling V Van Dulmen S Eide H. cross-sectional study on person-centred communication in the care of older people: the COMHOME study protocol. Br Med J Open. (2015) 5:7864. 10.1136/bmjopen-2015-007864 25877282 PMC4401848

[4] Williams KN Perkhounkova Y Jao YL Bossen A Hein M Chung S. Person-centered communication for nursing home residents with dementia: four communication analysis methods. West J Nurs Res. (2018) 40:1012–31. 10.1177/0193945917697226 28335698 PMC5581294

[5] Deane WH Fain JA. Incorporating Peplau's theory of interpersonal relations to promote holistic communication between older adults and nursing students. J Holist Nurs. (2016) 34:35–41. 10.1177/0898010115577975 25854267

[6] Christensen SS Wilson BL Edelman LS. Can I relate? a review and guide for nurse managers in leading generations. J Nurs Manag. (2018) 26:689–95. 10.1111/jonm.12601 29380917

[7] Mohile SG Epstein RM Hurria A Heckler CE Canin B Culakova E. Communication with older patients with cancer using geriatric assessment: a cluster-randomized clinical trial from the National Cancer Institute Community Oncology Research Program. J Am Med Assoc Oncol. (2020) 6:196–204. 10.1001/jamaoncol.2019.4728 31697365 PMC6865234

[8] Serafin L Danilewicz D Chyla P Czarkowska-Paczek B. What is the most needed competence for newly graduated generation z nurses? focus groups study. Nurse Educ Tod. (2020) 94:104583. 10.1016/j.nedt.2020.104583 32920466

[9] Sundler AJ Hjertberg F Keri H Holmström IK. Attributes of person-centred communication: a qualitative exploration of communication with older persons in home health care. Int J Older People Nurs. (2020) 15:12284. 10.1111/opn.12284 31642182

[10] Ministerio de Ciencia e Innovación. Orden CIN/2134/2008, de 3 de julio, por la que se establecen los requisitos para la verificación de los tí*tulos universitarios oficiales que habiliten para el ejercicio de la profesión de Enfermero* (2008). Available at: https://www.boe.es/boe/dias/2008/07/19/pdfs/A31680-31683.pdf (accessed May 25, 2022).

[11] Khodabandeh-Shahraki S Abazari F Pouraboli B Dehghan-Nayeri N. Communication behaviors in nursing homes in South-East Iran: an ethnographic study. Iran J Nurs Midwifery Res. (2019) 24:137–43. 10.4103/ijnmr.IJNMR_101_18 30820226 PMC6390432

[12] Anderson L. A Taxonomy for Learning, Teaching, and Assessing: a Revision of Bloom's Taxonomy of Educational Objectives. Boston, MA: Allyn&Bacon (2013).

[13] Ko E Kim HY. Effects of simulation-based education combined team-based learning on self-directed learning, communication skills, nursing performance confidence and team efficacy in nursing students. J Kor Acad Nurs. (2017) 24:39–50. 10.7739/jkafn.2017.24.1.39

[14] Bandura A. Guide for constructing self-efficacy scales. In:Pajares F Urdan T, editors. Self-Efficacy Beliefs of Adolescents. Charlotte, NC: Information Age Publishing (2006). p. 309.

[15] Singh A Dangmei J. Understanding the generation Z: the future workforce. South-Asian J Multidiscipl Stud. (2016) 3:5280948.

[16] Houghton CE Casey D Shaw D Murphy K. Staff and students' perceptions and experiences of teaching and assessment in Clinical Skills Laboratories: interview findings from a multiple case study. Nurse Educ Tod. (2012) 32:e29–34. 10.1016/j.nedt.2011.10.005 22078867

[17] Shorey S Kowitlawakul Y Devi MK Chen HC Soong SKA Ang E. Blended learning pedagogy designed for communication module among undergraduate nursing students: a quasi-experimental study. Nurse Educ Tod. (2018) 61:120–6. 10.1016/j.nedt.2017.11.011 29197264

[18] Choi H Lee U Jeon YS Kim C. Efficacy of the computer simulation-based, interactive communication education program for nursing students. Nurse Educ Tod. (2020) 91:104467. 10.1016/j.nedt.2020.104467 32464566

[19] Bosse HM Nickel M Huwendiek S Schultz JH Nikendei C. Cost-effectiveness of peer role play and standardized patients in undergraduate communication training approaches to teaching and learning. BMC Med Educ. (2015) 15:4–9. 10.1186/s12909-015-0468-1 26498479 PMC4619415

[20] Gillette C Stanton RB Rockich-Winston N Rudolph M Anderson HG. Cost-effectiveness of using standardized patients to assess student-pharmacist communication skills. Am J Pharm Educ. (2017) 81:73–9. 10.5688/ajpe6120 29367775 PMC5774195

[21] MacLean S Kelly M Geddes F Della P. Use of simulated patients to develop communication skills in nursing education: an integrative review. Nurse Educ Tod. (2017) 48:90–8. 10.1016/j.nedt.2016.09.018 27741440

[22] Judd M. Broken communication in nursing can kill: teaching communication is vital. Creat Nurs. (2013) 19:101–4. 10.1891/1078-4535.19.2.101 23798248

[23] Liebrecht C Montenery S. Use of simulated psychosocial role-playing to enhance nursing students' development of soft skills. Creat Nurs. (2016) 22:171–5. 10.1891/1078-4535.22.3.171 29195526

[24] Beaird G Nye C Thacker LR. The use of video recording and standardized patient feedback to improve communication performance in undergraduate nursing students. Clin Simul Nurs. (2017) 13:176–85. 10.1016/j.ecns.2016.12.00526154864

[25] Hashimoto H. Effects of a support program on nurses communication with hospitalized childrens families. Compr Child Adolesc Nurs. (2017) 40:173–87. 10.1080/24694193.2017.1307473 28749229

[26] Shao YN Sun HM Huang JW Li ML Huang RR Li N. Simulation-based empathy training improves the communication skills of neonatal nurses. Clin Simul Nurs. (2018) 22:32–42. 10.1016/j.ecns.2018.07.003

[27] MacLean S Kelly M Geddes F Della P. Evaluating the use of teach-back in simulation training to improve discharge communication practices of undergraduate nursing students. Clin Simul Nurs. (2018) 22:13–21. 10.1016/j.ecns.2018.06.005

[28] Furnes M Kvaal KS Høye S. Communication in mental health nursing - bachelor Students' appraisal of a blended learning training programme - an exploratory study. BMC Nurs. (2018) 17:1–11. 10.1186/s12912-018-0288-9 29785174 PMC5952371

[29] Cortés-Rodríguez AE Roman P López-Rodríguez MM Fernández-Medina IM Fernández-Sola C Hernández-Padilla JM. Role-play versus standardised patient simulation for teaching interprofessional communication in care of the elderly for nursing students. Healthcare. (2022) 10:10046. 10.3390/healthcare10010046 35052210 PMC8775804

[30] Connolly M Perryman J McKenna Y Orford J Thomson L Shuttleworth J. THYMETM: a model for training health and social care professionals in patient-focussed support. Patient Educ Couns. (2010) 79:87–93. 10.1016/j.pec.2009.06.004 19628353

[31] INACSL Standards Committee. INACSL standards of best practice: smulationSM debriefing. Clin Simul Nurs. (2016) 12:S21–5. 10.1016/j.ecns.2016.09.008

[32] Phrampus PE O'Donnell J. Debriefing using a structured and supported approach. In:Levine AI DeMaria S Schwartz AD Sim A, editors. The Comprehensive Textbook of Healthcare Simulation. New York, NY: Springer (2014). p. 73–84.

[33] Cortés-Rodríguez AE. Comparison of the Effects of Two Educational Strategies on the Development of Competence in Basic Professional Communication Amongst Nursing Students. (Ph. D. Thesis). University of Almería, Almería, Spain (2020).

[34] Hernández-Padilla JM Correa-Casado M Granero-Molina J Cortés-Rodríguez AE Matarín-Jiménez TM Fernández-Sola C. Psychometric evaluation and cultural adaptation of the Spanish version of the Scale for End-of Life Caregiving Appraisal. Palliat Support Care. (2019) 17:314–21. 10.1017/S1478951518000470 30073939

[35] Hernández-Padilla JM Suthers F Granero-Molina J Fernández-Sola C. Effects of two retraining strategies on nursing students' acquisition and retention of BLS/AED skills: a cluster randomised trial. Resuscitation. (2015) 93:27–34. 10.1016/j.resuscitation.2015.05.008 26026776

[36] Domuracki K Wong A Olivieri L Grierson LEM. The impacts of observing flawed and flawless demonstrations on clinical skill learning. Med Educ. (2015) 49:186–92. 10.1111/medu.12631 25626749

[37] Hernández-Padilla JM Granero-Molina J Márquez-Hernández V V Cortés-Rodríguez AE Fernández-Sola C. Effects of a simulation-based workshop on nursing students' competence in arterial puncture. Acta Paulista de Enfermagem. (2016) 29:678–85. 10.1590/1982-0194201600095

[38] The European Parliament and the Council of the European Union. Regulation (EU) 2016/679 of the European Parliament and of the Council Of 27 April 2016 on the Protection of Natural Persons With Regard to the Processing of Personal Data and on the Free Movement of Such Data, and Repealing Directive 95/46/EC (general da) (2016). p. 119/1–119/88.

[39] Bortolato-Major C Perez J Mattei Â Mantovani M de F Cestari JV Boostel R. Contributions of the simulation for undergraduate nursing students. J Nurs UFPE/Revista de Enfermagem UFPE. (2018) 12:1751–62. 10.5205/1981-8963-v12i6a230633p1751-1762-2018

[40] Quail M Brundage SB Spitalnick J Allen PJ Beilby AJ. Student self-reported communication skills, knowledge and confidence across standardised patient, virtual and traditional clinical learning environments. BMC Med Educ. (2016) 16:1–12. 10.1186/s12909-016-0577-5 26919838 PMC4769506

[41] Younis JR Mabrouk SM Kamal FF. Effect of the planned therapeutic communication program on therapeutic communication skills of pediatric nurses. J Nurs Educ Pract. (2015) 5:109–20. 10.5430/jnep.v5n8p109

[42] Aebersold M Tschannen D Sculli G. Improving nursing students' communication skills using crew resource management strategies. J Nurs Educ. (2013) 52:125–30. 10.3928/01484834-20130205-01 23380022

[43] Becker KL Rose LE Berg JB Park H Shatzer JH. The teaching effectiveness of standardized patients. J Nurs Educ. (2006) 45:103–11. 10.3928/01484834-20060401-03 16629278

[44] Claramita M Tuah R Riskione P Prabandari YS Effendy C. Comparison of communication skills between trained and untrained students using a culturally sensitive nurse-client communication guideline in Indonesia. Nurse Educ Today. (2016) 36:236–41. 10.1016/j.nedt.2015.10.022 26586255

[45] Khan BA Ali F Vazir N Barolia R Rehan S. Students' perceptions of clinical teaching and learning strategies: a Pakistani perspective. Nurse Educ Today. (2012) 32:85–90. 10.1016/j.nedt.2011.01.016 21333417

[46] Brown CE Back AL Ford DW Kross EK Downey L Shannon SE. Self-assessment scores improve after simulation-based palliative care communication skill workshops. Am J Hosp Palliat Med. (2016) 35:45–51. 10.1177/1049909116681972 28273752

[47] Hsu LL Chang WH Hsieh SI. The effects of scenario-based simulation course training on nurses' communication competence and self-efficacy: a randomized controlled trial. J Prof Nurs. (2015) 31:37–49. 10.1016/j.profnurs.2014.05.007 25601244

[48] Doyle D Copeland HL Bush D Stein L Thompson S. A course for nurses to handle difficult communication situations. A randomized controlled trial of impact on self-efficacy and performance. Pat Educ Couns. (2011) 82:100–9. 10.1016/j.pec.2010.02.013 20303230

[49] Cadorin L Suter N Dante A Williamson SN Devetti A Palese A. Self-directed learning competence assessment within different healthcare professionals and amongst students in Italy. Nurse Educ Pract. (2012) 12:153–8. 10.1016/j.nepr.2011.10.013 22112861

[50] Chen MC. Relationships among self-directed learning, learning styles, learning strategies and learning achievement for students of Technology University in Taiwan by using structural equation models. Recent Res Educat Technol. (2010) 2010:67–72.

[51] Lee J Mast M Humbert J Bagnardi M Richards S. Teaching handoff communication to nursing students: a teaching intervention and lessons learned. Nurse Educ. (2016) 41:189–93. 10.1097/NNE.0000000000000249 26866731

[52] Hagemeier NE Hess R Hagen KS Sorah EL. Impact of an interprofessional communication course on nursing, medical, and pharmacy students' communication skill self-efficacy beliefs. Am J Pharm Educ. (2014) 78:1–10. 10.5688/ajpe7810186 25657373 PMC4315208

[53] Rask MT Jensen ML Andersen J Zachariae R. Efeitos de uma intervenção visando melhoria na comunicação enfermeiro-paciente em um ambulatório de oncologia. Cancer Nurs. (2009) 32:1–11. 10.1097/01.NCC.0000343365.13871.12 19104195

[54] Annonio D Hoffman LA Zedreck J Robertson LB Tuite PK. Ready, SET, go improving patient-nurse communication. Nurs Manage. (2016) 47:40–9. 10.1097/01.NUMA.0000480760.76675.8f 26914385

[55] Curtis JR Back AL Ford DW Downey L Shannon SE Doorenbos AZ. Effect of communication skills training for residents and nurse practitioners on quality of communication with patients with serious illness: a randomized trial. J Am Med Assoc. (2013) 310:2271–81. 10.1001/jama.2013.282081 24302090 PMC4310457

[56] Schlegel C Woermann U Shaha M Rethans J-J van der Vleuten C. Effects of communication training on real practice performance: a role-play module versus a standardized patient module. J Nurs Educ. (2012) 51:16–22. 10.3928/01484834-20111116-02 22085207

[57] Doherty C Landry H Pate B Reid H. Impact of communication competency training on nursing students' self-advocacy skills. Nurse Educ. (2016) 41:252–5. 10.1097/NNE.0000000000000274 27175831

[58] Valadares AF Magro MC. Opinion of nursing students on realistic simulation and the curriculum internship in hospital setting. ACTA Paulista de Enfermagem. (2014) 27:138–43. 10.1590/1982-0194201400025

[59] Brydges R Nair P Ma I Shanks D Hatala R. Directed self-regulated learning versus instructor-regulated learning in simulation training. Med Educ. (2012) 46:648–56. 10.1111/j.1365-2923.2012.04268.x 22691145

[60] Roh YS Ahn JW Kim E Kim J. Effects of prebriefing on psychological safety and learning outcomes. Clin Simul Nurs. (2018) 25:12–9. 10.1016/j.ecns.2018.10.001

